# Supplementary material for: Biphasic Force-Regulated Phosphorylation Site Exposure and Unligation of ERM Bound with PSGL-1: A Novel Insight into PSGL-1 Signaling via Steered Molecular Dynamics Simulations
Source: Int J Mol Sci. 2020 Sep 25;21(19):7064. doi: 10.3390/ijms21197064 (PMC7583015; doi:10.3390/ijms21197064)
Supplement: Supplementary file 1 [file ijms-21-07064-s001.zip › Supplementary Materials/Description of Supplementary Materials.doc]

**Biphasic force-regulated phosphorylation site explosure and unligation of ERM bound with PSGL-1: a novel insight into PSGL-1 signaling via steered molecular dynamics simulations**

Jingjing Feng, Yan Zhang, Quhuan Li, Ying Fang and Jianhua Wu

**1, Figure S1**

Supplementary Information

**Figure S1.** The ensemble workflow of computational procedure.

**2, Figure S2**

 **Figure S2.** Structural stability of the complex being loaded with constant tensile force. The time curves of mean RMSD (**A**) and mean relative extension ΔL (**B**) of PSGL-1/FERM complex under tensile forces of 0.0, 25, 50,75 and 100 pN. The data were the mean of the results from three runs. (**C**) Gaussian fitting of the frequency distribution of interface H-bonds number in different time periods under different constant forces.

It was found from Figure S2 that, the C_α_ root mean square deviation (RMSD) of the clamped complex increased obviously with tensile force and slowly with the relaxing time, illustrating a redistribution of the structural unbalanced-strain built-up at the force-ramp stage (Figure S2-A); and, *ΔL*, the relative extension of the clamped subdomain of the complex, might be negligible for its values smaller than 0.8 Å, reflecting the conservation of the seven-stranded β-sandwich and the C-terminal α-helix of FERM domain C under loading of constant force (Figure S2-B and Figure 6; Materials and Method). We carried out Gaussian fitting for the frequency distribution of the number of H-bonds on binding site in different time periods during the force-clamp simulation, and found that the frequency distribution tended to be normal for sufficient sampling time > 100 ns (Figure S2-C), suggesting that sampling of 100 ns is suitable in this study.

**3, Figure S3**

**Figure S3.** Time courses of SASA for ITAM-like motif (red), phosphorylation site Y205 (blue) and Y192 (green) in force-clamp simulation under constant tensile forces. SASA values of the ITAM-like motif (**A to E**) and Y205 (**F to J**) increased significantly as time passed through times of about 25 ns, but SASA values of Y191 remain a very low level in comparison with Y205 (K to O). All data shown are the average of three run results.

The time courses of the averaged solvent-accessible surface area (SASA) for ITAM-like motif and its two phosphorylation sites Y205 and Y191 were plotted in Figure S3, which showed that, at zero tensile force, the SASA of the ITAM-like motif climbed from a low pseudo-plateau of about 650 Å^2^ to a higher one of about 700 Å^2^ at simulation time of 45 ns; but, as increasing of simulation time, the SASA of the ITAM-like motif fluctuated around the level of either about 700 Å^2^ for the tension of 25 pN or about 750 Å^2^ for tension of 100 pN, climbed quickly to a higher plateau of about 850 Å^2^ within 40 ns at tension of 50 pN, or increased quickly first and then decline to a lower plateau of about 700 Å^2^ at tension of 75 pN. Being excited by thermal fluctuation of molecules, the rigid-body motion of FERM C domain relative to FERM A and B domains should be responsible for the biplateau’s SASA-time pattern at zero tensile force (Figure S3-A), and, together with the tension-induced allostery of FERM C domain, contribute to the various SASA-time patterns of the ITAM-like motif under nonzero tensile forces (Figure S3-B,C,D and E). As time passed through, the SASA of Y205 would fluctuate around a level of about 50 Å^2^ at zero tensile force (Figure S3-F), but increase slowly with fluctuation at tensions of 25, 50, 75 and 100 pN (Figure S3-G,H,I and J), in consistent with the RMSD of the clamped complex (Figure S3-A), suggesting that the tension-induced allostery rather than the rigid-body motion of FERM C domain contributed to exposing of phosphorylation site Y205. The SASA value of the Y191 remained a low level of about 6 Å^2^ (Figure S3-K,L,M,N and O), because the phosphorylation site Y191 are buried in FERM B domain, which was beyond the force transduction pathway in the Loading strategy (Materials and Method).

Table S1. H-bonds survival ratios in PSGL-1/ERM complexes

| No | Residue name | | Force(pN) | | | | |
| --- | --- | --- | --- | --- | --- | --- | --- |
|  | PSGL-1 | FERM | 0 | 25 | 50 | 75 | 100 |
| 1 | Y410 | I248 | 0.98±0.01 | 0.96±0.04 | 0.98±0.01 | 0.98±0.01 | 0.98±0.01 |
| 2 | Y410 | H288 | 0.60±0.09 | 0.31±0.21 | 0.37±0.22 | 0.30±0.16 | 0.10±0.05 |
| 3 | Y410 | N247 | 0.21±0.00 | 0.09±0.00 | 0.07±0.02 | 0.11±0.00 | 0.18±0.11 |
| 4 | H408 | F250 | 0.84±0.15 | 0.89±0.04 | 0.90±0.06 | 0.88±0.09 | 0.76±0.17 |
| 5 | P417 | R295 | 0.62±0.00 | 0.31±0.18 | 0.69±0.14 | 0.58±0.00 | 0.25±0.00 |
| 6 | P417 | K296 | 0.19±0.08 | 0.57±0.00 | 0.19±0.00 | 0.00±0.00 | 0.00±0.00 |
| 7 | P417 | R246 | 0.00±0.00 | 0.18±0.01 | 0.19±0.00 | 0.61±0.32 | 0.49±0.24 |
| 8 | P417 | N247 | 0.00±0.00 | 0.00±0.00 | 0.07±0.00 | 0.13±0.00 | 0.23±0.00 |
| 9 | V412 | R246 | 0.49±0.29 | 0.49±0.02 | 0.60±0.10 | 0.52±0.15 | 0.30±0.18 |
| 10 | R413 | S243 | 0.47±0.05 | 0.19±0.15 | 0.25±0.27 | 0.38±0.17 | 0.19±0.08 |
| 11 | R413 | I245 | 0.37±0.01 | 0.18±0.07 | 0.37±0.19 | 0.15±0.12 | 0.08±0.00 |
| 12 | R413 | R246 | 0.22±0.03 | 0.35±0.18 | 0.18±0.06 | 0.27±0.05 | 0.27±0.16 |
| 13 | R413 | H288 | 0.11±0.00 | 0.10±0.02 | 0.38±0.01 | 0.14±0.00 | 0.06±0.00 |
| 14 | R405 | D252 | 0.43±0.32 | 0.70±0.10 | 0.73±0.02 | 0.64±0.16 | 0.66±0.15 |
| 15 | T407 | N251 | 0.26±0.13 | 0.23±0.06 | 0.23±0.06 | 0.17±0.10 | 0.20±0.03 |
| 16 | K406 | N251 | 0.06±0.01 | 0.34±0.46 | 0.09±0.03 | 0.08±0.01 | 0.08±0.03 |

The residual interaction indices in PSGL-1/ERM complexes were presented as means ±S.D. of three different residual interaction survival ratios measured from three independent simulations within simulation time of 100 ns for every force.

**Vedios S1 to S3.** To examine pull-induced unbinding of PSGL-1 from FERM, we performed the force-ramp SMD simulation over 30 ns thrice with time step of 2 fs and a pulling velocity of 5 Å/ns. The C-terminal Cα atom of FERM (S313) was fixed, and the N-terminal Cα atom of PSGL-1 (R402) was steered along pulling direction perpendicular to the binding surface of the complex. The virtual spring, connecting the dummy atom and the steered atom, had a spring constant of 69.48 pN/nm. Run 1, Run 2, and Run 3 in Figure 3 correspond to Vedio S1, Vedio S2, and Vedio S3, respectively.

**Vedios S4 to S8.** To further examine the regulation of tensile force on exposing of the phosphorylation sites of FERM bound with PSGL-1 in the force-clamp mode, the so called “ramp-clamp” SMD simulations were performed thrice for tensile forces of 25, 50, 75 and 100 pN. Once tensile force *f* arrived at a given value, such as 25, 50, 75 or 100 pN, the SMD simulation was transformed from the force-ramp mode to a force-clamp one, at which the complex was stretched with the given constant tensile force for the followed 100 ns. Each events of hydrogen bonding under stretching were recorded to examine the involved residues and their functions. However, the stretching of 0 pN is derived from equilibrium simulation, 0, 25, 50, 75 or 100 pN correspond to Vedio S4, Vedio S5, Vedio S6, Vedio S7, and Vedio S8, respectively.
